# Supplementary material for: Are carbon emissions trading and green financial instruments synergistic? -Comprehensive quantitative research based on content analysis
Source: PLoS One. 2024 Mar 7;19(3):e0298601. doi: 10.1371/journal.pone.0298601 (PMC10919601; doi:10.1371/journal.pone.0298601)
Supplement: S1 Data — (ZIP) [file pone.0298601.s001.zip › Data/地级市Z_F工作报告.zip!/▒▒╛⌐/└┤╫╘╣1⁄2╓┌║┼í╛┬φ┐╦╩2╛▌═°í┐_2014.docx]

──2014年1月16日在北京市第十四届人民代表大会第二次会议上

北京市市长　王安顺

各位代表：

　　现在，我代表北京市人民政府，向大会报告政府工作，请予审议，请市政协各位委员提出意见。

　　一、过去一年工作回顾

　　2013年，是全面贯彻落实党的十八大精神的第一年，伟大中国梦激起全市人民团结奋斗的无比信心和力量。面对经济下行压力较大、人口资源环境矛盾凸显的复杂形势，在党中央、国务院和中共北京市委的坚强领导下，全市人民同心同德、脚踏实地、迎难而上，经济社会发展取得新的成就，实现了良好开局。全市地区生产总值比上年增长7.7%；地方公共财政预算收入3661.1亿元，增长10.4%；城镇居民人均可支配收入超过4万元，农村居民人均纯收入超过1.8万元，分别实际增长7.1%和7.7%；万元地区生产总值能耗、水耗均下降4%左右，大气主要污染物排放量下降3%以上，较好地完成了市十四届人大一次会议确定的各项目标任务。

　　一年来，我们认真学习贯彻党的十八大、十八届二中、三中全会和习近平总书记一系列重要讲话精神，深入开展党的群众路线教育实践活动，坚持稳中求进，坚持依法行政，直面矛盾问题，深化改革开放，主要做了以下工作：

　　(一)加快转变经济发展方式，促进结构调整和优化升级，提高经济增长的质量和效益。坚持创新驱动，落实先行先试各项政策，中关村示范区企业总收入突破3万亿元、增长20%以上，全市技术合同成交额增长16%。促进服务业提质增效，金融业、科技服务业、商务服务业增加值分别增长11%、11.2%和9.5%，规模以上文化创意产业收入增长7.5%左右，服务业企业利润增长20%左右。加快重点功能区和重大产业项目建设，六大高端产业功能区增加值在全市经济中的比重提高到44%左右，规模以上高技术制造业、现代制造业增加值分别增长10.4%和14.2%。加强投资项目调度，出台促进电子商务发展等系列促消费政策，举办北京购物季、惠民文化消费季等活动，全社会固定资产投资增长8.8%，社会消费品零售额增长8.7%，内需潜力稳定释放。

　　(二)全面推进首都生态文明建设，下决心治理大气污染，努力改善城乡环境。制定实施2013-2017年清洁空气行动计划，完成3428蒸吨燃煤锅炉清洁能源改造，实施核心区4.4万户煤改电工程，更新老旧机动车36.6万辆，退出污染企业288家，压缩水泥产能150万吨，农村地区换用优质煤36万吨、减少用煤44万吨。开展工地扬尘、渣土运输、河湖水环境等专项治理行动，建立城市环境考核评价体系，综合整治城乡环境脏乱死角。发挥政府投资杠杆作用，吸引社会投资18亿元，新增垃圾日处理能力3000吨，新建续建污水处理、再生水利用设施29个，污水处理率中心城达到96.5%、郊区达到61%以上，再生水利用量8亿立方米。坚决治理违法建设，全年拆除1261.4万平方米，初步实现了新生违法建设“零增长”。完成平原造林36.4万亩，新增城市绿地1100公顷，林木绿化率、森林覆盖率分别达到57.4%和40%。成功举办第九届园博会，树立了绿色发展的新典范。

　　(三)坚持建设与治理并重，着力解决突出问题，增强城市运行保障能力。持续推进轨道交通建设，4条新线投入运营，通车里程达到465公里，在建里程208公里。完成道路微循环改造和疏堵工程139项，开通92条商务班车和袖珍公交线路，新增居住区和地下停车位4.1万个，采取一系列措施缓解“打车难”问题。积极调整能源结构，西南热电中心、陕京三线、大唐煤制气一期等重点工程建成投用，优质能源比重提高到77%。完成20座下凹式立交桥积水改造，治理中小河道280公里，建成一批雨洪利用设施，实现了城市安全度汛。严格市场秩序监管，查处取缔各类无证无照经营2.1万户。全面开展安全生产大检查，强化综合监管和行业监管，安全生产事故起数和死亡人数双下降。从源头预防和减少社会矛盾，持续推进突出治安问题专项整治，社会大局保持安全稳定。

　　(四)注重发展的整体性与协同性，全力推进各项重点工作，促进城乡区域一体化发展。深化农村各项改革，完成了集体土地所有权确权，开展存量用地建设公租房试点，农民专业合作社达到6010家，集体经济组织产权制度改革继续深化。完成8万户农宅抗震节能改造，小城镇建设和新型农村社区试点取得新成效。加强城市副中心和新城建设，启动实施第二阶段城南行动计划，积极落实西部地区转型发展重大项目，持续推进城乡结合部城市化改造。促进生态涵养发展区可持续发展，建成一批特色精品沟域，实施1.48万山区农民搬迁。

　　(五)顺应人民群众新期待，加强和创新社会管理，不断改善人民群众生活。投入财政资金196.8亿元，32件惠民实事全面落实。完成学前教育三年行动计划，增加幼儿园学位3万个。落实中小学建设三年行动计划，新增城乡一体化学校30所、学位2.4万个，实施减负八条措施，减轻了学生课业负担。稳步推进市属公立医院改革试点，社会举办的医疗机构新增86家。扩大基本药物目录和社区药品医保报销范围，总额预付试点医院增加到196家，城乡居民大病保险二次报销上不封顶。完善覆盖城乡的公共文化服务体系，深入实施文化惠民工程，群众文化体育蓬勃发展。就业、物价形势稳定，年末城镇登记失业率为1.21%，全年居民消费价格上涨3.3%。出台了加快养老服务业发展的意见。实施城乡统一的招用安置政策，将灵活就业的农村劳动力纳入城镇职工社会保险制度，社会保障相关待遇标准提高10%左右。保障性住房建设超额完成任务，建设16.2万套、竣工8.5万套、配租配售4.7万套。启动中心城110项棚户区改造工程，完成1390万平方米老旧小区综合改造，推出2万套共有产权性质的自住型商品住房。进一步加强社会建设，四类社会组织实现直接登记，社会服务管理网格化体系覆盖到70%以上的社区和村庄。

　　(六)坚持改革开放不动摇，加大重点领域和关键环节改革力度，增强经济社会发展活力。推进政府职能转变和机构改革，调整完善了食品药品监管体制，组建了卫生计生委和新闻出版广电局，完成了劳教制度改革任务。两次开展行政审批事项清理，市级审批事项由1562项减少至940项，减少了39.8%。开展优化投资项目审批流程试点，审批时限由300多个工作日缩短到109个工作日。国有企业改革进展顺利，市属企业实现利润增长22%。

　　放开市政基础设施领域社会资本投资，推出了引资规模达1300亿元的126个试点项目。“营改增”有序推进，试点企业总体税负下降41.2%。积极推进出租车、垃圾处理、排污和非居民用热、用气等价格调整。加快发展要素市场，碳排放权交易开市运行，全国中小企业股份转让系统挂牌公司达到356家。

　　开放型经济体系进一步完善。克服外需不足的困难，地区进出口总额增长5.1%，服务贸易总额增长20%以上，实际利用外资85.2亿美元，境外直接投资超过24亿美元。接待旅游人数2.5亿人次，旅游总收入达到3963.2亿元。与天津、山西、河北签订合作框架协议，开展高层次对接协商，推动区域交通、农业、科技等领域务实合作，协同建立京津冀及周边地区大气污染防治协调机制，区域合作发展迈入新阶段。坚持首善标准，圆满完成对口支援年度任务。

　　各位代表，一年来，我们高度重视民主法制和精神文明建设，军政军民团结、妇女儿童事业、残疾人事业都取得了卓有成效的进展，民族、宗教、外事、对台和侨务工作进一步加强，和谐社会首善之区的氛围更加浓厚。

　　这些成绩的取得，是党中央、国务院和中共北京市委坚强领导，市人大、市政协和社会各界支持帮助，全市人民奋力拼搏的结果。我们真切感受到，人民群众是真正的英雄，北京建设发展取得的一切成就，都是人民群众创造的。在这里，我代表北京市人民政府，向辛勤工作、无私奉献的全市各族人民，表示崇高的敬意！向给予政府工作大力支持的人大代表、政协委员、各民主党派、各人民团体和各界人士，向中央各部门、各兄弟省区市，向驻京解放军和武警部队官兵，向关心支持北京建设发展的港澳台同胞、海外侨胞和国际友人，表示衷心的感谢！

　　我们清醒地认识到，经济社会发展和政府工作还存在不少问题。一是人口资源环境压力加大。人口数量增长过快，中心城区功能疏解缓慢，优质公共资源过于集中，大气污染、垃圾污水治理任务艰巨，城市可持续发展面临挑战。二是经济发展结构性矛盾突出。科技、文化资源优势没有充分发挥出来，产业内部结构不合理，投资对房地产的依赖度还比较高，一些不符合首都城市功能定位的产业退出缓慢，区县同质化发展现象突出。三是城市服务管理水平不高。城乡基础设施的承载力与公共需求还不适应，市区交通拥堵仍比较严重，背街小巷、城乡结合部环境治理滞后，小广告、露天烧烤、“黑车”等痼疾顽症久治不愈；生产安全、食品药品安全、公共安全领域还存在不少问题。四是关系群众切身利益的问题亟待解决。义务教育发展不均衡，基层公共文化设施不完善，保障房建设供给不足，棚户区居住条件较差，一些群众生活不够便利。五是从严治政需要不断加强。政府职能转变还不到位，重审批、轻监管现象仍然存在，一些部门工作不扎实、效率不高；整治“四风”，加强勤政廉政建设，还要狠下功夫。我们将高度重视，不以事艰而不为，不以任重而畏惧，采取有力措施，切实加快解决，让人民群众见到实实在在的成效。

　　二、2014年主要任务

　　进入新时期新阶段，北京已经成为现代化国际大都市，经济社会发展活力、综合竞争力、国际影响力持续增强。但在长期快速发展中，也积累形成了比较明显的“城市病”，解决体制性结构性矛盾，缓解发展不平衡、不协调、不可持续的问题更为迫切。面对新时期首都发展的形势和任务，我们必须准确把握战略定位，深刻认识阶段性特征，咬定目标不放松，解决问题不懈怠，探索创新特大城市可持续发展的体制机制，努力把首都建设好、发展好。

　　第一，坚持把改革创新贯穿于经济社会发展各个领域各个环节，从制约首都可持续发展最突出的问题入手，从群众期盼、社会关注的热点难点问题改起，建立健全市场起决定性作用、政府更好发挥作用的资源配置体系和首都功能调控体系，让发展之路越走越宽。

　　第二，坚持把转变经济发展方式摆在首要位置，不单纯以地区生产总值定政绩，从首都城市性质、功能定位和资源优势出发，围绕提高经济发展的质量效益，充分发挥科技创新、文化创新的支撑引领作用，加强规划引导和统筹协调，持续优化调整产业结构，率先形成创新驱动、城乡一体的发展新格局。

　　第三，坚持把法治作为治理城市的基本方式，创新社会治理，发挥好政府的主导作用，尊重人民群众的主体地位，鼓励和支持社会各方面参与，加强法治保障，严格依法行政，运用法治思维和法治方式化解社会矛盾，提高城市精细化管理水平，维护首都安全稳定。

　　第四，坚持把群众满意作为政府工作的第一标准，牢固树立全心全意为人民服务的根本宗旨，持续推进基本公共服务均等化，努力解决好就业、社保、教育、医疗、养老、住房等重要民生问题，维护社会公平正义，保障人民安居乐业。

　　2014年是全面贯彻落实党的十八届三中全会精神、全面深化改革的第一年，改革任务繁重艰巨，外部环境复杂多变。政府工作的总体要求是：高举中国特色社会主义伟大旗帜，以马克思列宁主义、毛泽东思想、邓小平理论、“三个代表”重要思想、科学发展观为指导，深入贯彻落实党的十八大、十八届三中全会、中央经济工作会议、中央城镇化工作会议和习近平总书记一系列重要讲话精神，按照市委十一届三次、四次全会部署，坚持稳中求进、改革创新，着力激发发展活力，着力破解城市发展难题，着力强化创新驱动，着力推进新型城镇化，着力保障和改善民生，着力加强精细化管理，推动首都经济持续健康发展，保持社会和谐稳定。

　　全市经济社会发展主要预期目标是：地区生产总值增长7.5%左右；城镇登记失业率控制在2%左右；城镇居民人均可支配收入、农村居民人均纯收入均实际增长7.5%左右；居民消费价格涨幅控制在4%左右；地方公共财政预算收入增长9%；常住人口增长速度明显下降；万元地区生产总值能耗下降2%以上、水耗下降4%以上，二氧化碳排放量下降2.5%以上，大气细颗粒物年均浓度下降5%左右。

　　围绕实现上述目标，要着重抓好以下几个方面的工作：

　　(一)着力激发发展活力，全面深化改革

　　进一步解放思想，认真研究、系统筹划各项改革，抓好重大改革措施的细化落实，加快推进已明确方向的改革任务，深化正在进行的改革事项，推动经济社会更有效率、更加公平、更可持续发展。

　　加快转变政府职能。进一步理顺政府与市场的关系，简政放权，减少政府对微观事务的管理，加强公共服务、市场监管、社会管理、环境保护职责。继续取消、下放审批事项，规范保留的审批事项。推广优化投资项目审批流程试点经验，建立统一的网上审批平台，加快建设市级政务服务中心，创新审批服务机制，提高审批效率。严格控制机构编制，财政供养人员只减不增。制定政府购买服务的实施意见，促进公共服务供给多元化。积极稳妥推动事业单位分类改革，推进有条件的事业单位转企改制。

　　坚持和完善基本经济制度。完善国有资产管理体制，探索由管企业为主向管资本为主转变，明确国有资本投资运营的方向和重心，逐步建立分类监管模式。深化国有资本控股经营的自然垄断行业改革，推进公共资源配置市场化。推动国有企业完善现代企业制度，加快股份制改革和上市步伐。积极发展混合所有制经济，支持市属国有企业与央企、民营、集体、外资等各类企业融合发展。支持非公有制经济健康发展，依法保护各种所有制经济产权与合法权益，制定非公有制企业进入特许经营领域的具体办法。落实促进中小企业发展条例，完善中小企业公共服务体系，及时解决民营企业和中小微企业发展中的问题，不断优化发展环境。

　　完善现代市场体系。推动工商注册制度便利化，统筹准入管理与事中、事后管理，对妨碍统一市场和公平竞争的各种规定进行清理，创新市场监管方式，抓紧健全社会征信体系，建立全市统一的市场主体信用信息平台，完善信用披露制度和信用约束机制。加快建设多层次资本市场，支持中小企业股份转让系统扩容，推动区域性股权交易市场发展。改革完善土地储备制度，推行工业用地弹性出让和租赁制，逐步建立城乡统一的建设用地市场。进一步理顺非居民用水、用气、用电价格，推行居民生活用水阶梯价格制度。按照经营类、公益类分开原则，做好公用事业补贴管理改革工作，适时推进公共交通票制票价改革。

　　加大财税体制改革力度。完善预算管理制度，探索跨年度预算平衡机制，推动编制三年滚动预算，加强支出管理，调整支出结构，提高财政资金使用效益。健全债务管理制度，规范债务“借、管、用、还”，逐步将政府性债务纳入全口径预算管理，并报同级人民代表大会审查批准，防控债务风险。推进“营改增”扩围，对接好国家消费税、资源税等税制改革。合理划分市与区县两级事权和支出责任，完善一般性财政转移支付制度，清理、整合、规范专项转移支付项目，增加对大气污染防治、河道治理、环境整治项目的投入。健全大额专项资金动态调整机制，扩大市级预算绩效管理试点范围，开展财政支出竞争性分配试点，完善财政预决算信息公开制度。

　　深化投融资体制改革。依据首都城市性质和功能定位，研究制定负面清单，确立企业投资主体地位。完善投资运营和回报补偿机制，鼓励支持社会资本投资，在轨道交通、城市道路、综合交通枢纽、污水处理、固废处理、镇域供热等领域继续推出一批试点项目，拓展棚户区改造、古都风貌保护融资渠道，在教科文卫体等领域积极引入社会资本，推动非基本公共服务领域市场化、社会化。规范完善政府投融资平台管理和融资机制，争取开展地方政府发债试点。建设科技金融服务平台，推进国家保险产业园建设，支持民间资本依法发起设立中小型银行、金融租赁公司、消费金融公司等金融机构，促进互联网金融健康发展，建立地方金融安全监管体系。

　　进一步深化农村改革。坚持和完善农村基本经营制度，深入推进土地流转起来、资产经营起来、农民组织起来，赋予农民更多财产权利，促进城乡要素平等交换、公共资源均衡配置。加快构建新型农业经营体系，鼓励承包土地经营权向新型经营主体流转，推进农用地承包经营权、集体建设用地使用权确权颁证，建立农村产权流转交易市场。推广大兴西红门、海淀东升经验，开展统筹利用集体建设用地试点，发展壮大集体经济。完善农村金融服务体系，加强农业风险防范制度建设。深化集体林权制度改革。探索闲置农民住房有组织盘活利用。开展征地制度改革试点，推进征地农转非和整建制农转居，使农民成为拥有集体资产的市民。

　　健全绿色发展的体制机制。完善生态文明建设的考核指标体系和考核机制，建立最严格的环境保护监管制度，完善生态补偿和资源有偿使用制度，加大对生态保护区的支持力度。积极推动森林、绿地、湿地、河湖水系等生态保护红线的划定工作，严格保护自然生态空间。研究建立资源环境承载能力监测预警机制，对饮用水源保护区、生态敏感脆弱地区、环境质量严重超标地区实行限制性开发措施。支持开展碳汇交易试点，建立社会资本投入生态环保的市场化机制。

　　创新社会治理体制。坚持系统治理、依法治理、综合治理、源头治理，不断提升社会治理水平，实现政府治理和社会自我调节、居民自治良性互动。完善社区居委会、村委会组织体系和运行机制，推行“参与式协商”自治模式，拓宽居民参与社会治理的范围和途径。继续完善“枢纽型”社会组织工作体系，培育一批高质量的社会组织，实行行业协会、商会与行政机关脱钩，探索一业多会。加强社会工作者队伍建设，推进志愿服务制度化。统筹开展社区、社会组织、社工联动试点，增强基层社会治理能力。深入开展法制宣传教育，加强法律服务队伍建设，实现法律服务社区村全覆盖，提高市民法治意识，推动建设法治城市。

　　(二)着力破解城市发展难题，不断提高可持续发展水平

　　人口资源环境矛盾是现阶段躲不开、绕不过的发展难题，关系人民群众切身利益，关系首都形象，关系发展全局，必须严肃面对、标本兼治。

　　加强人口规模调控。从落实城市功能定位、优化产业结构、调控资源配置、加强规划引导等方面入手，深入研究控制人口规模的治本之策。立足当前，着眼长远，细化落实各项具体措施，切实把常住人口增速降下来。一是加强统筹协调。对重大规划、重大政策、重大项目进行人口评估和交通评价、水资源评价，研究建立与人口调控挂钩的政府投资、公共资源分配机制，明确区县调控责任，实行重点督查考核。二是抓好产业调控。修订产业准入目录，强化节能节地节水、环境、技术、安全等标准，完善税收征管，推进服务业优化升级，严控低端产业无序发展，严厉打击违法建设和非法生产经营。三是强化以房管人。支持制定房屋租赁条例，推广组织化管理模式，依法管理出租房屋，继续治理群租房问题。四是改进服务管理。落实“单独两孩”生育政策，积极研究相关服务保障措施。做好流动人口基础登记办证工作，建设实有人口服务管理全覆盖体系，推行居住证制度。

　　全力治理大气污染。认真落实大气污染防治条例，扎实推进各项治理工程，确保完成清洁空气行动计划年度任务。改造燃煤锅炉4100蒸吨，基本取消五环路以内的燃煤锅炉房，推进新一轮农村电网改造，加快治理城乡结合部和农村地区原煤散烧，全面建成四大燃气热电中心，全年削减燃煤260万吨，退出污染企业300家。压缩小客车年度配置指标，逐年增加新能源汽车比重，基本淘汰黄标车。严管施工现场和渣土运输，大幅减少扬尘污染。继续抓好大尺度森林和森林廊道建设，基本完成平原百万亩造林主体任务，做好森林和绿地湿地的管护工作。加快推进气象现代化，开展细颗粒物成因研究和减排关键技术攻关，抓好重污染日应急预案实施。加强宣传教育和舆论引导，发挥全社会参与、支持、监督的积极性。

　　着力推进垃圾污水治理。全面实施三年行动计划，加快垃圾、污水处理设施建设，完成100处非正规垃圾填埋场治理，全市污水处理率达到85%，利用再生水8.6亿立方米。落实污染源头治理责任，加强企业污染源监管，创新河道流域管理模式和联合执法工作机制，严肃查处违法违规排污行为。健全垃圾处理体系和全过程管理体系，积极采用焚烧、综合处理和资源化技术，推进生活垃圾分类和源头减量，提高建筑垃圾资源化利用水平。

　　增强资源能源保障能力。坚持最严格的水、土地资源管理制度，加强资源能源利用效率评价。全面推进南水北调配套工程，抓紧实施水系联通和水资源战略储备工程，改造300公里中心城区供水管线，确保汛后安全通水。加快构建综合防洪排涝体系，完成500公里中小河道疏浚和23座立交桥积水治理，抓好雨洪利用和农田水利工程建设。加强能源供应的调度和储备，增强供气、供电保障能力。实施6500万平方米住宅供热计量改造，对全市3000平方米以上的公共建筑设置耗电限额，积极发展绿色建筑。推广应用节水节能新产品、新技术，在全社会形成节约光荣、浪费可耻的良好风尚。

　　深入推进区域合作发展。落实国家区域发展战略，积极配合编制首都经济圈发展规划，抓紧编制空间布局、基础设施、产业发展和生态保护专项规划，建立健全区域合作发展协调机制，主动融入京津冀城市群发展。提高市场化合作水平，发挥企业主体作用，促进区域资金、人才、信息、技术等各种要素的优化配置。着力推进基础设施互联互通，抓好京沈客运专线、京唐客运专线、京张城际铁路、京昆高速北京段、国道110二期等重点项目，促进交通一体化。健全大气污染联防联控合作机制，协同推进京津风沙源治理、三北防护林等重点项目建设，整体构建区域生态环境屏障。继续推进对口支援和帮扶协作，促进优势互补、共同发展。

　　(三)着力强化创新驱动，加快推进经济结构调整

　　下足“进”的功夫，做好“退”的文章，加快培育以技术、品牌、服务为核心竞争力的新优势，增强发展的协调性和可持续性。

　　用科技创新推动北京新发展。加快科技体制改革，打通科技与经济社会发展之间的通道，最大限度释放科技生产力。一是提升中关村示范区整体创新能力。统筹谋划科技创新综合改革，挖掘先行先试系列政策潜力，研究制定新一轮创新发展支持政策，完善一区多园统筹发展机制，出台示范区优化产业布局指导意见，推进中关村科学城、未来科技城、科技商务区建设。二是让企业成为技术创新的主体。健全企业主导技术创新的体制机制，实施企业研发机构建设等三项工程，支持企业与高校院所开展协同创新。更加注重培育市场化的科技服务组织，引导科技企业孵化器、创新型孵化器集聚发展，为企业创新提供专业化服务。三是把更多科技成果转化为现实生产力。实施技术创新行动计划，加快推进关键核心技术研发和应用。创新科研成果转化和收益分配制度，全方位深化央地合作、院市合作、军民融合，完善部市会商工作机制，加强产业专项资金使用统筹，推动一批重大科技成果产业化项目落地。四是构建更为灵活的人才引进和使用机制。扩大科技开放与合作，实施海内外人才引进专项计划，支持各类人才工作室、创业基地建设。完善科技项目经费管理机制，提高经费使用自主权，增强对关键岗位、核心骨干的激励。五是实施首都知识产权和标准化战略，健全市场导向、激励奖励、转化应用、打击侵权等四项机制，加强知识产权保护和运用。做好世界知识产权组织办事处落户服务工作。

　　激发文化创造活力。坚持社会主义先进文化前进方向，树立高度的文化自觉和文化自信，培育和践行社会主义核心价值观。深化文化管理体制改革，健全国有文化资产监管机制，完善文化事业单位法人治理结构，推进转制单位建立现代企业制度，加快公司制、股份制改造，鼓励社会资本参与国有文化企业改制重组。制定文化创意产业功能区建设发展规划，抓好核心演艺区、文化保税区、国家数字出版基地等重点项目，促进新兴业态发展。制定现代公共文化服务体系建设的实施办法，推进区域文体中心建设，改善社区文化活动条件，实施好文化惠民工程，推动基本公共文化服务标准化、均等化。扩大文化资助和文化采购，鼓励创作精品力作，为群众提供更多更好的精神文化产品和服务。

　　统筹推进差异化特色化发展。突出人口规模、发展质量、生态环境等三个方面，建立区县差异化考核评价指标体系，降低首都功能核心区、生态涵养发展区经济增长指标的考核权重。完善重大产业项目统筹协调机制，强化各级各类园区土地节约集约利用评价。抓好商务中心区核心区建设，加快金融街内涵式发展和资源置换，推动北京经济技术开发区产业提升、产城融合，完善奥林匹克中心区整体功能，支持临空经济区转型升级，增强六大高端产业功能区集聚度和承载力。积极推进四个高端产业新区重大基础设施建设，聚集培育主导产业。深入实施第二阶段城南行动计划，促进西部地区转型发展。加快生态涵养发展区重点沟域建设，继续做好山区农民搬迁工作，建设32条生态清洁小流域，全面建成雁栖湖生态示范区，扎实推进世界园艺博览会筹备工作，带动旅游休闲等绿色产业发展。

　　深度调整产业内部结构。遵循特大城市产业发展规律，构建与首都功能定位相适应的现代产业体系，促进三次产业融合发展。做强有优势、有基础的现代制造业，原则上不再发展一般制造业。积极发展教育、科技、文化、卫生、体育等领域的非基本公共服务，提升金融、信息、商务等生产性服务业发展水平。努力培育新一代信息技术、生物、新能源等领域的新增长点，发展壮大战略性新兴产业。抓住大规模环境建设的契机，做大节能环保产业。加快发展新能源汽车产业，做好充换电设施的规划建设，鼓励购买新能源汽车。把握大数据时代特征，用互联网思维改造提升传统产业，推动移动互联网、物联网、云计算等产业加快发展。认真做好第三次经济普查。落实与中央单位、驻京部队的合作协议，切实将首都资源优势转化为发展优势。

　　优化内需结构。统筹用好政府投资，注重提高投资效益，更多投向环境治理、民生改善和基础设施建设，更多投向农村和发展薄弱地区。加强投资促进，做好各类市场主体的投资服务，提高民间投资比重。合理安排建设节奏，抓好搬迁与建设、主体与配套、公益性建设与经营性开发的衔接，加快重大项目建设。发挥消费的基础性作用，促进商品消费稳定增长，大力发展服务消费，积极引导绿色节能消费，加快发展电子商务、网络消费，扩大信息消费规模。深化国家旅游综合改革试点，支持旅游产业发展，增强旅游服务能力，提升外来消费和旅游消费水平。出台优化消费环境实施意见，加强企业诚信经营监管，让市民安全放心消费。

　　发展开放型经济。发挥“京交会”的带动作用，扩大文化贸易，提升服务贸易发展水平。加快建设外贸转型示范基地和跨境电子商务产业园，创建优势产品出口质量示范区，鼓励拥有自主品牌和自主知识产权的企业扩大出口。推动海关特殊监管区的整合优化，落实贸易投资便利化措施，增强口岸服务功能。提高利用外资质量，支持企业开拓国际市场，推动设计咨询、文化、品牌等“走出去”。深化与港澳台地区经贸合作，做好外事、对台和侨务工作。积极与河北省协调配合，共同申办第24届冬奥会。

　　(四)着力推进新型城镇化，率先形成城乡发展一体化新格局

　　坚持把新型城镇化作为治理“城市病”、实现城乡一体化的重要抓手，修改城市总体规划，推进经济发展规划、城乡建设规划、土地利用规划“三规合一”，划定城市开发边界，优化城乡功能和空间布局，坚决扭转城市发展“摊大饼”，提升城镇化质量。

　　推进中心城功能疏解。强化规划指导和控制，严格控制中心城建设规模和开发强度，除重点功能区和棚户区改造项目外，对房地产开发和公共服务项目就地扩建，设置更高的限制条件。加强配套政策研究，制定商品交易市场管理办法，引导服装、小商品、建材市场逐步退出。研究中心城优质公共资源和人口向新城疏解的利益引导机制，完善企业跨区县迁移管理办法，促进资源要素合理配置与生产力布局优化。加强历史文化名城保护，加大文保区保护修缮力度，延续城市历史文脉。

　　增强城市副中心和新城承载能力。把副中心、新城作为发展重点，提供更好的工作机会和生活环境，尽快建设成为优化城市布局的重要载体。编制通州城市副中心到2020年的发展规划，完成核心启动区重大基础设施主体工程，引进建设一批优质教育、医疗、文体等公共服务项目。拓展大兴—亦庄、顺义、昌平、房山等综合性新城功能，完善连接中心城区的快捷交通体系，集聚符合发展方向的产业和服务要素。建设好门头沟、怀柔、平谷、密云、延庆等区域性新城，强化基础设施和产业支撑，推动职住平衡，带动区域发展。推动新机场主体工程全面开工，加快外围交通体系建设，筹划建设新机场临空经济区。

　　积极稳妥推进农村城镇化。坚持以人为核心，统筹城乡结合部地区、小城镇和新型农村社区建设。做好50个重点村后续建设发展，深化第一道绿化隔离地区6个乡镇城市化建设试点，并逐步推开。加强乡镇统筹，以盘活存量为主，提高建设用地集约化水平，力争4到5年明显改善城乡结合部地区面貌。梯次推进小城镇建设，鼓励与大型企业、高校对接共建，大力发展特色产业，促进农村人口就近城镇化。严格规划和用途管制，严肃查处违法建设、销售“小产权房”。抓好48个新型农村社区试点建设，将村级组织公益事业补助标准提高50%，完成8万户农宅抗震节能改造，完善农村基础设施和公共服务设施长效管护机制。传承乡土文化，保持农村风貌，推进旧村整治更新，提升人居环境，建设美丽乡村。

　　提高农村经济发展水平。自觉落实粮食生产责任，抓好“米袋子”、“菜篮子”重点工程，建立基本农田补贴制度，加强基本农田保护，实施农业基础建设及综合开发二期工程，增加储备规模，保持一定的粮食和蔬菜自给率。转变农业发展方式，加快建设国家现代农业科技城，做强现代种业，办好世界种子大会、世界葡萄大会，大力发展资源节约型、环境友好型农业，提升都市型现代农业发展水平。坚持源头治理、标本兼治，用最严谨的标准、最严格的监管、最严厉的处罚、最严肃的问责，确保首都农产品质量安全。落实农村经济薄弱地区发展规划，支持低收入村提升集体经济发展能力。大力培养新型农民，推进绿色就业，提高公益性岗位补助标准，引导农民转移就业，积极帮扶低收入农户增收，保持农民收入快速增长。

　　(五)着力保障和改善民生，不断增进人民群众福祉

　　“天地之间，莫贵于人”。面对人民群众过上更好生活的期盼，我们要持续加大民生投入，认真办好惠民实事，提供更加均衡的基本公共服务，织好保障民生安全网。

　　满足群众基本住房需求。深入研究住房建设规律，构建以政府为主提供基本保障、以市场为主满足多层次需求的住房保障和供应体系。一是坚持以公租房和共有产权房为重点，理顺建设管理体制，建设保障性住房7万套。加快推进已开工项目，年内竣工10万套，确保“十二五”期间提供100万套，逐步解决本市城镇中低收入家庭住房困难。二是加快推进城六区棚户区改造和环境整治，2017年基本完成四环路以内的棚户区改造任务，年内同步完成1000万平方米老旧小区综合改造。三是推进共有产权性质的自住型商品住房建设，加快已供地项目实施进度，新增5万套供地，优化住房供应结构。四是继续严格执行房地产调控各项政策措施，坚决保持房价基本稳定，促进房地产市场平稳健康发展。加强和创新房屋租赁市场管理，保障出租人、承租人合法权益。

　　努力为市民健康服务。深化基层医疗卫生机构综合改革，巩固完善基本药物制度，开展全科医生制度试点，抓好中医药服务提升工程，增强基层医疗卫生服务能力。制定实施区域卫生规划，加强医疗卫生资源整合，健全社区首诊、分级就诊、双向转诊机制，规范就医秩序。完善市属公立医院改革试点配套政策，调整医疗服务价格，逐步扩大改革试点范围。落实社会办医18条政策，推进一批社会办医项目落地。推动天坛医院、口腔医院等大医院迁建。深化医保付费方式改革，提升医保基金管理水平。建设全市统一的医疗卫生信息平台。加强健康促进工作，完善全民健身公共服务体系，提高市民健康水平。

　　办好人民满意的教育。全面贯彻党的教育方针，坚持立德树人，形成爱学习、爱劳动、爱祖国活动的长效机制，增强学生社会责任感、创新精神、实践能力。强化体育课和课外锻炼，促进青少年身心健康。制定实施促进义务教育优质均衡发展的政策措施，加大对农村地区的教育投入，新增20所城乡一体化学校，提高农村地区中小学办学质量。推进课程、教学、考试评价整体改革，出台中考中招、高考高招改革框架方案。健全教师培养培训和流动机制，完善义务教育学校校长教师交流轮岗制度，深化绩效工资和职称评定改革。继续抓好公立幼儿园建设，扶持普惠型民办幼儿园发展。支持高中特色校建设。扩大中高职衔接办学改革试点，培养首都发展需要的技工人才。实施高等教育协作发展计划，促进高校办出特色、争创一流。

　　全力促进就业增收。完善城乡统一的就业创业政策，全面做实就业服务、就业管理、就业援助工作，扩大职业技能培训，大力帮扶城乡困难群体就业，城镇新增就业36万人。做好高校毕业生就业促进工作，加强离校未就业毕业生的就业指导和帮扶，健全鼓励到基层工作的服务保障机制，完善小额担保贷款等相关政策，增强青年群体创新创业活力。继续深化收入分配制度改革，加快建立合理的收入增长机制，增加城乡居民特别是低收入群体收入。

　　提升社会保障水平。坚持保基本、兜底线、促公平、可持续，完善职工基本养老保险与城乡居民养老保险的衔接办法，稳步提高各项社会保障待遇标准。加快社会救助城乡统筹，完善医疗救助制度，探索建立“支出型”贫困救助制度，扩大救助范围。健全困境儿童等特殊人群的福利政策，提高优抚保障水平，鼓励发展慈善事业。健全基本养老服务制度，完善支持政策，积极推动养老、健康服务业发展，建设80个街道乡镇养老照料中心，建立一批小型社区养老机构，推行居家养老助残服务卡，发展“医养结合”服务。

　　切实保障妇女和未成年人权益，健全残疾人社会保障和服务体系。推进民族团结进步事业发展，认真贯彻党的宗教工作方针，欢迎海外侨胞和归侨侨眷参与首都现代化建设。坚持走中国特色军民融合发展路子，加强全民国防教育，支持部队改革建设，做好征兵和转业安置工作，巩固发展军政军民团结。

　　(六)着力加强精细化管理，提升城市服务保障水平

　　坚持精细化、人性化和智能化的城市管理理念，积极创新服务管理方式，努力解决突出问题，让城市更加宜居、社会更加有序、生活更加便利。

　　提高城乡环境质量。全面开展城乡环境综合整治行动，重点抓好旅游景区、繁华商业区、进京通道等30个区域和204条道路的环境达标建设，集中整治背街小巷、城乡结合部、区县交界等地区的脏乱死角。保持打击违法用地违法建设高压态势，严明纪律，严格执法，确保新生违法建设“零增长”，拆除存量违建1000万平方米。重拳治理环境秩序，对小广告、露天烧烤、“黑车”等城市顽疾，实施分级分类和全过程管理。改善群众身边环境，加快城市休闲公园建设，新增1000公顷城市绿地，建成200公里市级绿道。深化规划设计，完善设施配套，逐步解决建成小区存在的问题。

　　持续改善市民出行条件。落实公交优先战略，加快轨道交通建设，新增通车里程62公里，研究编制到2020年的轨道交通建设规划。完善城市快速路和主干路系统，改造中心城主要拥堵节点，两年内完成200公里道路微循环畅通工程。构建地面公交快速通勤系统，开拓多样化地面公交服务，抓好交通枢纽场站和驻车换乘设施建设，中心城公交出行比例达到48%。提升农村客运服务水平，方便农村特别是偏远山区居民出行。合理规划整治行人步道和自行车道，加快建设公共自行车租赁系统。实施停车综合改革，开展老旧居住区停车自治管理试点，坚决治理乱停车。加强交通秩序管理，完善分区域、分时段限行措施，做好交通信息预测预报，引导车辆合理出行。

　　加强城市服务管理。深化城市管理体制改革，推动管理重心下移、专业职能下沉，做实街乡、做强社区，加快城市管理网格、社会服务网格、社会治安网格有序对接，提升基层服务管理能力。建成200个“一刻钟社区服务圈”，使总数突破1000个、覆盖到60%的城市社区，让群众在家门口就能享受到10大类60项基本公共服务，以及多样化的便利服务。建立地下管网综合管理体制，落实行业主管部门责任，提升城市运行监控和预警应急能力，保障水电气热供应和安全运行。实施“宽带北京”行动计划，完善信息化基础设施，加快建设“智慧城市”。

　　三、巩固发展教育实践活动成果，加强政府自身建设

　　全力维护首都安全稳定。坚持管行业、管业务、管生产经营必须同时管安全，深化安全生产管理体制改革，建立隐患排查治理体系和安全预防控制体系，开展地铁、群租房、地下空间、特种设备、城乡结合部等重点领域和地区专项整治行动，坚决遏制重特大事故发生。加强食品药品安全工作，完善工作体系，健全覆盖生产、经营、消费全过程的监管制度。加强应急体系软硬件建设，建立防灾减灾救灾综合管理体制，提高突发事件的应对处置能力。深化信访工作改革，畅通诉求表达渠道，落实领导干部接访下访制度，完善人民调解与行政调解、司法调解衔接联动工作机制，加强法律援助，提升基层排查化解矛盾纠纷的能力。加大依法监管力度，维护网络和信息安全。深化“平安北京”建设，把反恐防暴纳入维稳工作体系，创新立体化社会治安防控体系，突出群防群治、专群结合，提高社会治安整体管控能力，营造和谐稳定的社会环境。

　　2014年，亚太经合组织领导人非正式会议将在北京举行。做好服务保障工作，是继奥运会、残奥会和新中国成立60周年庆祝活动之后，中央交给我们的又一项光荣任务，也是对全市各项工作的一次全面检验。要高度重视、全力以赴，加快主会场建设，确保5月底前完成场馆及配套工程建设任务，改造市区相关会议设施设备，认真开展运营调试，确保万无一失。细化接待服务、交通调度、安全保障等方案预案，提供高水平、特色化服务。制定实施环境保障方案，做好环境布置和绿化美化工作，展现整洁有序、清新亮丽的城市形象。发挥人民群众主人翁精神，深入开展精神文明创建活动，提升城市文明水平和市民文明素质，让服务保障的成果不断巩固、惠及全市人民。

　　过去一年，我们认真落实党的群众路线教育实践活动部署，严格遵守中央八项规定和北京市15条实施意见，努力把“为民、务实、清廉”贯彻到政府工作的方方面面。教育实践活动中，各方面提出的174项意见建议，已经立行立改119项，纳入工作计划、稳步推进解决的55项；市政府召开的常务会、专题会、全市性大会减少40.4%，以市政府名义举办的活动减少了50%，市级部门“三公”经费等一般性支出压缩1.27亿元。

　　新的一年，我们要慎终如始，以踏石留印、抓铁有痕的劲头，继续抓好各项整改措施的落实，不断提高政府公信力和执行力，全面建设法治政府和服务型政府。

　　坚持科学民主决策。以群众利益、群众需求作为决策的基本出发点，认真听取各方面对政府工作的意见和建议。完善重大行政决策程序，坚持调研论证，落实信访评估、社会稳定风险评估、行政决策风险评估“三评合一”。邀请群众代表列席市政府涉及民生议题的会议，完善公众参与、专家咨询和政府决策相结合的机制。涉及重大公共利益、人民群众切身利益的决策事项，都要向社会公开征求意见或举行听证会。深化政务信息公开，推行公共服务办事公开制度，方便群众了解政府工作、监督政府工作。

　　坚持依法行政。严格按照法定权限和程序行使权力、履行职责，坚决执行市人大及其常委会的决议和决定，坚持重大事项报告制度，自觉接受人大工作监督、法律监督和人民政协民主监督，完善协商民主制度，认真听取各民主党派、工商联、无党派人士和人民团体的意见建议，自觉接受人民群众和新闻舆论监督。制定推进依法行政、建设法治政府的工作规划，完善依法行政考核评价制度。做好政府立法工作，推动轨道交通安全、建筑工程质量、环境秩序、人口管理等方面立法。理顺城管执法体制，整合执法力量和协管队伍，完善综合执法、行政与司法之间的协调配合机制，加强执法监督检查，全面落实执法责任制和执法过错责任追究制。加强行政复议工作，认真做好行政应诉。

　　坚持从严治政。加强公务员特别是领导干部的教育管理，狠抓政风行风建设，组织开展明察暗访，让群众和服务对象进行评议，持续治理“庸懒散”，完善差别化的绩效管理，提高行政效能和服务水平。推行权力清单制度，依法公开权力运行流程，强化对政府职能部门履行职责情况的监督，加强行政监察和审计监督，加大行政问责力度。深入学习贯彻十八届中央纪委三次全会精神，严格执行党风廉政建设责任制，健全廉政风险防控体系，加大违法违纪案件查办力度，旗帜鲜明地反对腐败，确保廉洁从政。严格执行《党政机关厉行节约反对浪费条例》、《党政机关国内公务接待管理规定》，坚持勤俭办一切事业，“三公”经费支出预算降低12%以上，市级节庆论坛展会活动的财政经费压缩40%以上。

　　坚持求真务实。切实增强问题意识、进取意识、机遇意识、责任意识，依靠群众，发动群众，勇往直前，攻坚克难。紧紧围绕首都发展重大问题，加强调查研究，察实情、出实招、办实事，总结基层经验，推动工作落实。坚持“两个务必”，坚决整治“四风”，以新的实际行动保持同人民群众的血肉联系。牢记使命，恪尽职守，夙夜在公，始终保持干事创业的热情、动真碰硬的豪情、奋发有为的激情，不断创造经得起实践、群众和历史检验的业绩，取信于民、造福于民。

　　各位代表！梦想承载希望，奋斗开创未来。让我们更加紧密地团结在以习近平同志为总书记的党中央周围，高举中国特色社会主义伟大旗帜，在中共北京市委坚强领导下，团结依靠全市人民，胸怀中国梦，积聚正能量，敢于担当，真抓实干，谱写首都改革开放和现代化建设的新篇章！

　　名词解释

　　社会服务管理网格化体系：是借鉴城市网格管理理念基础上的一种创新工作体系，它充分运用电子地图和现代信息技术，通过在一定行政服务管理区域科学划分网格，把区域内人、地、物、事、组织等社会服务管理内容全部纳入网格，统筹整合各种社会服务管理资源和力量，及时有效发现和解决社会服务管理问题，实现社会服务管理信息化、智能化和精细化。

　　城市病：是对人口及相关发展要素向大城市过度集聚而引起的一系列社会管理和公共服务问题的统称。我国一些特大城市已经出现了不同程度的城市病，主要表现为人口过多、交通拥堵、房价高涨、环境恶化等。

　　大气细颗粒物：是指空气动力学直径小于等于2.5微米(约为人类纤细头发直径的1/20-1/30)的颗粒物，又称为可入肺颗粒物，是表征大气环境质量的主要污染物指标。根据颗粒物英文(ParticulateMatter)，可简写为PM2.5。

　　政府购买服务：就是通过发挥市场机制作用，把政府直接向社会公众提供的一部分公共服务事项，按照一定的方式和程序，交由具备条件的社会力量承担，并由政府根据服务数量和质量向其支付费用。

　　一业多会：指允许按照国民经济行业分类设立多个协会组织，或者是按照产业链各个环节、经营方式或服务类型设立行业协会，并允许行业协会整合重组，适度竞争，激发工作活力。

　　物联网：即“物物相连的互联网”。是指通过信息传感设备，按照约定的协议，把物品与互联网连接起来，进行信息交换和通讯，以实现智能化识别、定位、跟踪、监控和管理的一种网络。

　　云计算：是一种商业计算模型。它将计算任务分布在大量计算机构成的资源池上，使各种应用系统能够根据需要获取计算力、存储空间和信息服务。提供资源的网络被称为“云”，“云”中的资源在使用者看来是可以无限扩展、随时获取的，并按使用付费。

　　亚太经合组织领导人非正式会议：亚太经合组织是亚太地区级别高、范围广、相对成熟的政府间经济合作机制，成立于1989年，现有21个成员，包括中、美、俄、韩等国家和台湾、香港两个经济体。在亚太经合组织领导人非正式会议期间(统称“领导人会议周”)，将举行领导人非正式会议、高官会、工商咨询理事会、工商领导人峰会等一系列会议和活动。其中领导人非正式会议是亚太经合组织最高级别的会议，主要就有关经济问题发表见解，凝聚共识，会议形成的领导人宣言是指导亚太经合组织各项工作的重要纲领性文件。1993年，首次领导人非正式会议在美国西雅图召开，此后每年召开一次。2012年9月在俄罗斯符拉迪沃斯托克举行的领导人非正式会议上，宣布中国将举办2014年亚太经合组织会议第二十二次领导人非正式会议，2013年5月，中央决定在北京举办。
